# Toward DNA-Based T-Cell Mediated Vaccines to Target HIV-1 and Hepatitis C Virus: Approaches to Elicit Localized Immunity for Protection

**DOI:** 10.3389/fcimb.2019.00091

**Published:** 2019-04-03

**Authors:** Zelalem A. Mekonnen, Branka Grubor-Bauk, Makutiro G. Masavuli, Ashish C. Shrestha, Charani Ranasinghe, Rowena A. Bull, Andrew R. Lloyd, Eric J. Gowans, Danushka K. Wijesundara

**Affiliations:** ^1^Virology Laboratory, Basil Hetzel Institute for Translational Health Research, Discipline of Surgery, University of Adelaide, Adelaide, SA, Australia; ^2^Molecular Mucosal Vaccine Immunology Group, The John Curtin School of Medical Research, The Australian National University, Canberra, ACT, Australia; ^3^Viral Immunology Systems Program, The Kirby Institute, The University of New South Wales, Sydney, NSW, Australia

**Keywords:** DNA vaccine, hepatitis C, human immunodeficiency virus, HIV/AIDS, HCV, tissue-resident memory, T cell immunity

## Abstract

Human immunodeficiency virus (HIV)-1 and hepatitis C virus (HCV) are major contributors to the global disease burden with many experts recognizing the requirement of an effective vaccine to bring a durable end to these viral epidemics. The most promising vaccine candidates that have advanced into pre-clinical models and the clinic to eliminate or provide protection against these chronic viruses are viral vectors [e.g., recombinant cytomegalovirus, Adenovirus, and modified vaccinia Ankara (MVA)]. This raises the question, is there a need to develop DNA vaccines against HIV-1 and HCV? Since the initial study from Wolff and colleagues which showed that DNA represents a vector that can be used to express transgenes durably *in vivo*, DNA has been regularly evaluated as a vaccine vector albeit with limited success in large animal models and humans. However, several recent studies in Phase I-IIb trials showed that vaccination of patients with recombinant DNA represents a feasible therapeutic intervention to even cure cervical cancer, highlighting the potential of using DNA for human vaccinations. In this review, we will discuss the limitations and the strategies of using DNA as a vector to develop prophylactic T cell-mediated vaccines against HIV-1 and HCV. In particular, we focus on potential strategies exploiting DNA vectors to elicit protective localized CD8^+^ T cell immunity in the liver for HCV and in the cervicovaginal mucosa for HIV-1 as localized immunity will be an important, if not critical component, of an efficacious vaccine against these viral infections.

## Introduction

Human immunodeficiency virus (HIV)-1 and hepatitis C virus (HCV) are significant contributors to the global disease burden with ~36.9 million people living with HIV-1 and at least 71 million people persistently infected with HCV (WHO, 2017; UNAIDS, 2018). Anti-retroviral therapy (ART) and direct acting anti-virals (DAAs) have contributed significantly to prolonging the lifespan and curing of HIV-1- and HCV-infected individuals, respectively (Cihlar and Fordyce, 2016; Zhang, 2016), but the annual HIV-1 and HCV incidences are still rising by millions. Furthermore, only 17 million (<50%) people have access to ART (Cihlar and Fordyce, 2016) and only 20% of patients are diagnosed for HCV (WHO, 2017). Additional issues involving drug resistance, reactogenicity associated with life-long ART and the lack of universal access to testing and cost-subsidized therapies minimize the ability of effective anti-viral drugs to end the HIV-1 and HCV epidemics. Thus, there is an urgent need to develop effective prophylactic vaccines to control the number of new infections and reduce the burden of supplying ART and DAA therapies to patients (Shin, 2016; Stone et al., 2016).

HIV-1 and HCV are rapidly mutating RNA viruses that exhibit considerable genetic diversity (nine subtypes in the major group of HIV-1 (German Advisory Committee Blood SAoPTbB., 2016) and 8 genotypes (gt1-8) of HCV which include at least 67 subtypes (Borgia et al., 2018) making immunity that develops during natural infection mostly ineffective. The lack of immune correlates of protection and convenient animal models permissive to infection make vaccine design and testing extremely challenging, and have also contributed to the fact that there is still no licensed vaccine for either HIV-1 or HCV (Wang et al., 2015; Bailey et al., 2019). HIV-1 and HCV co-infections represent an additional obstacle (Platt et al., 2016) although a recent clinical study suggests that co-administration of HIV-1 and HCV vaccines in humans can elicit robust HIV-1- and HCV-specific T cell responses without perturbing the immunodominance hierarchies of T cells responding against the vaccine encoded HIV-1 or HCV antigens (Hartnell et al., 2018).

DNA vaccines have been investigated for nearly three decades and are essentially bacteria-derived plasmids genetically engineered to encode immunogens under the control of promoters that facilitate robust expression of DNA in mammalian cells to induce adaptive immunity (Ferraro et al., 2011). DNA vaccines are inexpensive, easily constructed, stable at room temperature, replication defective in transfected mammalian cells and have minimum side effects which simplifies handling and distribution such that even developing countries can benefit from DNA vaccines (Jorritsma et al., 2016). Furthermore, plasmid DNA can be more easily used in multi-dose regimens unlike recombinant virus vectors that suffer from anti-vector immunity (Frahm et al., 2012). Recent seminal studies described therapeutic DNA vaccination against human papillomavirus (HPV) which resulted in histological regression and/or eliminated persistent HPV infection and HPV-related cervical lesions (Kim et al., 2014; Trimble et al., 2015). More recently, a DNA vaccine was developed that induced protective neutralizing antibodies (NAb) to Zika virus (ZIKV) in mice (Larocca et al., 2016) and rhesus macaques (Abbink et al., 2017) leading to the development of safe and immunogenic ZIKV DNA vaccines for humans (Tebas et al., 2017; Gaudinski et al., 2018). Thus, the many advantages of using plasmid DNA to develop vaccines and the recent developments of DNA vaccines in eliciting protective immunity in humans and higher animal models warrant further examination as to how DNA vaccines can be harnessed in vaccination regimens to target HIV-1 and HCV.

## Immune Targets for HIV-1 and HCV Prophylactic Vaccine Development

It is imperative that vaccines take into account the virus tropism, transmission routes, pathogenesis and immune responses that provide effective resistance against infections to elicit protective immunity against HIV-1 and/or HCV.

It is now established that mucosal tissues, mainly the gentio-rectal tissues and gastrointestinal tract, are the major sites of HIV-1 entry and pathogenesis, respectively (Belyakov and Ahlers, 2012). Induction of robust HIV-specific immune responses at these sites will be necessary to prevent HIV-1 infection or at the very least control viraemia during the acute phase of infection thus reducing the viral set point (McMichael and Koff, 2014) and infection-induced microbial translocation which can result in diversion of immune responses to counteract dysbiosis (Vujkovic-Cvijin et al., 2013). Furthermore, a prophylactic HIV-1 vaccine will likely be delivered using an active immunization strategy and attempt to mimic immune responses reported to be protective in macaques against simian immunodeficiency virus (SIV) and/or provide resistance against natural HIV-1 infections (Pontesilli et al., 1998; Saez-Cirion et al., 2007; Hansen et al., 2011; Haynes et al., 2012; Barouch et al., 2015, 2018; Ackerman et al., 2016; Borducchi et al., 2016). In this regard, the most protective immune responses reported to date involve T cell-mediated immunity (CMI) (Pontesilli et al., 1998; Saez-Cirion et al., 2007; Hansen et al., 2011; Borducchi et al., 2016), polyfunctional antibody responses (Barouch et al., 2015, 2018; Ackerman et al., 2016), antibody-dependent cellular cytotoxicity (Haynes et al., 2012), and broadly neutralizing antibodies (bNAb) (Burton and Hangartner, 2016). Although potent bNAb represent a blueprint for HIV-1 vaccine design, these antibodies are unlikely to be as effective in preventing cell to cell transmission compared to neutralizing cell free virus (Parsons et al., 2017). Consequently, a highly effective prophylactic HIV-1 vaccine will likely also rely on CMI to target highly conserved viral proteins such as Gag and Pol (Rolland et al., 2007) and/or non-neutralizing antibodies to broadly target the virus Envelope to prevent cell-cell transmission of the virus.

Unlike HIV-1 which has a relatively broad tropism, HCV is a bloodborne virus that primarily infects and replicates in hepatocytes. In primary hepatitis C infection, ~25% of patients naturally clear the virus and although reinfection occurs in many individuals (Grebely et al., 2012), it is evident that repeated infection is associated with a reduced magnitude and duration of viraemia, and a greater likelihood of clearance (Sacks-Davis et al., 2015). Thus, characterizing and eliciting the naturally-protective immune responses during primary infection and reinfection provide a rational path for the design of a prophylactic HCV vaccine (Grebely et al., 2012). The immune responses that correlate best with natural protection include robust and broad CMI to conserved HCV non-structural (NS) proteins (NS3, NS4, and NS5) (Smyk-Pearson et al., 2008; Baumert et al., 2014) and NAb targeting conserved regions of the viral envelope (E1E2) proteins (Houghton, 2011; Osburn et al., 2014; Bailey et al., 2017). Although CMI will not prevent infection, clinical data suggest that T cell responses could prevent the development of persistent infection in individuals who naturally clear the virus, which is an acceptable outcome given that primary infection is often asymptomatic and not associated with severe disease outcomes (Baumert et al., 2014). After two decades of unsuccessful pre-clinical studies and Phase I HCV vaccine trials, the current lead prophylactic candidate is in an NIH-sponsored Phase IIb, placebo-controlled trial (ClinicalTrials.gov Identifier: NCT01436357) in high risk people who inject drugs (PWID) (Swadling et al., 2014). The candidate vaccination regimen being tested utilizes a chimpanzee adenovirus (ChAd) prime and a modified vaccinia Ankara (MVA) boost to elicit systemic T cell immunity to gt1 NS antigens (Swadling et al., 2014). However, it is not clear if this vaccination can induce robust intrahepatic T cell immunity and sufficient multi-genotypic immunity to result in significant protection in vaccinated individuals especially given the increased prevalence of multiple genotypes in HCV endemic regions.

## T Cell-Mediated DNA Vaccines Against HIV-1 and HCV in the Clinic

DNA vaccines against HCV have been routinely tested in small and large animals including non-human primates (Latimer et al., 2014; Gummow et al., 2015; Grubor-Bauk et al., 2016; Wijesundara et al., 2018). Some candidates have also progressed in phase I/II clinical trials, but none have progressed to a large-scale efficacy trial in humans. A promising DNA vaccine that included a cocktail of four plasmids with each plasmid encoding codon optimized NS3/4A, NS4B, NS5A, or NS5B sequences from gt1a/b virus was used to prime/boost vaccinate macaques by electroporation (Latimer et al., 2014). In this study, the vaccine induced CD4^+^ and CD8^+^ T cells against each of the NS proteins encoded in the DNA cocktail which has resulted in the testing of the DNA cocktail in a phase I clinical trial (ClinicalTrials.gov Identifier: NCT02027116) although the results are yet to be disclosed.

A DNA vaccine has been tested for therapeutic vaccination against HCV. 12 hepatitis C patients suffering from chronic disease received three doses of a DNA vaccine encoding codon optimized NS3/4A from gt1a virus via electroporation on the deltoid muscle which induced NS3-specific CMI and a transient decrease in viral RNA levels (Weiland et al., 2013). The vaccine was also tested in eight patients who received interferon and ribavirin treatment of which six patients were completely cured of the infection (Weiland et al., 2013). Thus, DNA vaccines could be exploited in therapeutic settings against HCV, but this is unlikely to occur in the future given the success of using DAA to cure hepatitis C patients.

DNA vaccines against HIV-1 have been tested in different pre-clinical models and some have been tested in phase I/II clinical trials (Okuda et al., 1997; Cafaro et al., 2001; Tomusange et al., 2016). The first human clinical trial of a DNA vaccine, encoding *env* and *rev* genes, against HIV-1 was conducted in 1998 (MacGregor et al., 1998). Following vaccination of HIV-1 positive, treatment naïve individuals, no significant changes were observed in CD4^+^ and CD8^+^ T cell responses as well as in plasma HIV RNA. In another phase I clinical trial a DNA vaccine that encoded *env* and *rev* was shown to induce CD4^+^ T cell and poor CD8^+^ T cells responses in HIV-1 seronegative individuals (MacGregor et al., 2002). Similarly, low CD8^+^ T cell responses were observed in another phase I clinical trial following prime/boost vaccination with a DNA vaccine that encoded *gag* and *pol* genes (Tavel et al., 2007). More robust HIV-specific T cell responses have been elicited when DNA vaccines are used to prime and recombinant viral vectors are used to boost immune responses (Kibuuka et al., 2010; Bakari et al., 2011; Churchyard et al., 2011; Hayton et al., 2014; Moyo et al., 2017). However, prime/boost vaccinations with DNA vaccines alone can be optimized to elicit robust immune responses in humans against HIV-1. For instance, a retrospective study evaluating the immunogenicity of 10 HIV-1 DNA vaccine trials that used DNA vaccines in the absence of viral vectors or adjuvants suggest that the use of DNA delivery devices (e.g., electroporators and biojectors), and increasing the number of vaccine doses and dosage could more reproducibly elicit CD4^+^ and CD8^+^ T cell responses (Jin et al., 2015).

The main limitation associated with DNA vaccines is their inability to induce long-term immune responses following a single or a few vaccinations (Abbink et al., 2017). Furthermore, DNA vaccines are poorly effective and not well-optimized in eliciting immunity in the liver, gut or genito-rectal mucosa which warrant further refinements of DNA-based vaccination regimens in order to elicit durable protection against HIV-1 and/or HCV.

## The Potential of Tissue-Resident Memory T Cells For Controlling HIV-1 and HCV Infections

Since the initial discovery of highly cytotoxic memory T cells residing in tissues (Masopust et al., 2001), several studies have shown that CD8^+^ tissue-resident memory T (T_RM_) cells residing in the female reproductive tract, the gut, the lung and the liver form a formidable frontline defense against various pathogen infections (Mueller and Mackay, 2016; Rosato et al., 2017). The protective role of CD8^+^ T_RM_ cells is primarily due to their ability to (1) maintain a stable and durable population following their formation in tissues even in the absence of cognate antigen encounter following their formation (Gebhardt et al., 2009; MacKay et al., 2012; Beura et al., 2018; Park et al., 2018), and (2) produce anti-viral cytokines and/or exert cytotoxic functions to reduce the number of pathogen-infected cells and to recruit other immune cells (e.g., circulating memory T cells) rapidly to the site of infection (Schenkel et al., 2013; Muruganandah et al., 2018; Park et al., 2018). Furthermore, CD8^+^ T_RM_ cells respond more rapidly, produce greater amounts of anti-viral/cytotoxic molecules (i.e., in the liver) and appear to be crucial for protection against liver tropic pathogens and pathogens exposed in the vagina and the female reproductive tract compared to circulating memory T cells (Cuburu et al., 2012, 2015; Shin and Iwasaki, 2012; Fernandez-Ruiz et al., 2016; Beura et al., 2018). The greater frequency of intrahepatic CD8^+^ T_RM_ cells (CD69^+^ CD103^+^) amongst the total CD8^+^ T cell population correlated with partial control of viraemia in Hepatitis B Virus (HBV)-infected patients (Pallett et al., 2017), providing further encouragement that intrahepatic HCV-specific CD8^+^ T_RM_ cells will likely be protective against HCV.

**Figure 1:**
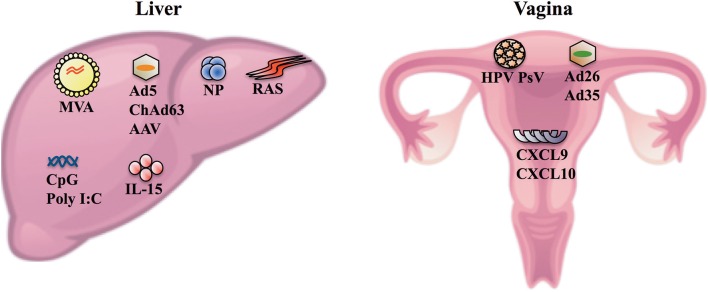
Vaccine vectors and agents that can be used to elicit and/or enhance the formation of CD8^+^ T_RM_ cells in the liver and the vagina. Following intravenous delivery, MVA, Ad serotype 5 (Ad5), ChAd serotype 63 (ChAd63), AAV, NP, and RAS can enter hepatocytes and/or cells surrounding the hepatic tissues (e.g., Kupffer cells) (Nganou-Makamdop et al., 2012; Tay et al., 2014; Gola et al., 2018). Intravenous delivery of these vectors in multi-dose prime/trap (Fernandez-Ruiz et al., 2016; Olsen et al., 2018), prime/target (Gola et al., 2018) or homologous prime/boost (Ishizuka et al., 2016) regimens elicits protection following *P. berghei* sporozoite challenge in mice or controlled *P. falciparum* infections in humans, and elicits intrahepatic CD8^+^ T_RM_ cells (Fernandez-Ruiz et al., 2016; Ishizuka et al., 2016; Gola et al., 2018). The prime/trap approaches involve priming CD8^+^ T cells systemically using antibodies that deliver a peptide antigen to cross-presenting dendritic cells (Fernandez-Ruiz et al., 2016) or using gene gun delivery of DNA encoding the cognate antigen (Olsen et al., 2018). The primed CD8^+^ T cells are then recruited to the liver and differentiate into T_RM_ cells (i.e., trapped) following intravenous delivery of AAV (Fernandez-Ruiz et al., 2016) or RAS (Olsen et al., 2018) that enter cells in the liver and express the relevant cognate antigen which are recognized by the primed CD8^+^ T cells. The prime/target approach is an adaptation of the prime/trap approach and essentially involves priming naïve CD8^+^ T cells with Ad5 or ChAd63 vaccine vector delivered via the intramuscular route and recruiting the primed CD8^+^ T cells to the liver following intravenous delivery of Ad5, NP, MVA, or ChAd63 vaccine vector (Gola et al., 2018). The same study showed that intravenous delivery was more efficient than intramuscular delivery of vaccine vectors to elicit high numbers of intrahepatic CD8^+^ T_RM_ cells and protection against *P. berghei* sporozoite challenge in the prime/target approach (Gola et al., 2018). IL-15 appears to be crucial for activated CD8^+^ T cells to differentiate into T_RM_ cells in the liver and inflammatory signals (e.g., CpG and Poly I:C) can enhance the formation of intrahepatic CD8^+^ T_RM_ cells *in vivo* (Holz et al., 2018). In the vagina, intravaginal delivery of HPV PsV, and more recently Ad26 and Ad35 have been shown to transduce cervicovaginal epithelial cells and elicit HPV-specific CD8^+^ T_RM_ cells in the cervicovaginal mucosa (Cuburu et al., 2012, 2015, 2018). In the absence of a vaccine vector, topical application of CXCL9 and CXCL10 in the vagina can be used to recruit/pull CXCR3^+^ effector CD8^+^ T cells into the vagina which subsequently differentiate into cervicovaginal T_RM_ cells (Shin and Iwasaki, 2012).

Despite HIV-1 and HCV being highly mutable with a complex and evolving quasispecies, several studies have revealed that only one or few variants, referred to as transmitted/founder (T/F) viruses, establish infection following transmission reflecting a strong genetic bottleneck (Bull et al., 2011; Joseph et al., 2015). T/F viruses will be exposed in the genito-rectal mucosa (i.e., the vagina and the rectum) during the vast majority (>80%) of HIV transmission and in the liver during HCV transmission. Thus, eliciting HIV- and HCV-specific CD8^+^ T_RM_ cells in the genito-rectal mucosa and the liver, respectively, following vaccination is also an attractive strategy to circumvent issues associated with viral diversity and eliminate these viruses shortly after transmission/exposure. Several vaccine vectors such as radiation attenuated sporozoites (RAS), protein loaded nanoparticles (NP), adenovirus (Ad) vectors, adeno-associated virus (AAV), and HPV pseudovirus (HPV PsV) have been developed to elicit localized protection and in some instances elicit CD8^+^ T_RM_ cells in the liver or the vagina (Figure 1) (Cuburu et al., 2012, 2015, 2018; Fernandez-Ruiz et al., 2016; Ishizuka et al., 2016; Gola et al., 2018). This provides hope that a vaccine to elicit intravaginal or intrahepatic CD8^+^ T_RM_ cells can be developed to potentially provide protection against HIV-1 or HCV, respectively.

A recent study suggests that strategies that can induce interleukin (IL)-15 and/or inflammation in the liver can be effective in recruiting circulating effector CD8^+^ T cells to differentiate into CD8^+^ T_RM_ cells in the liver (Holz et al., 2018). Systemic immunization strategies that promote up-regulation of gut homing molecules such as α4β7 on antigen-primed CD8^+^ T cells in secondary lymphoid organs can be efficiently recruited to establish residency in the gut (Masopust et al., 2010). Although these studies and others suggest that local antigen deposition and antigen encounter are not essential to elicit CD8^+^ T_RM_ cells, it is evident that this process leads to the formation of greatest densities of CD8^+^ T_RM_ cells especially at sites such as the liver and the vagina (Cuburu et al., 2012; Fernandez-Ruiz et al., 2016; Davies et al., 2017). Intravenous delivery of vaccine vectors appears to be most efficient route to facilitate local, intrahepatic expression of vaccine encoded antigens (Nganou-Makamdop et al., 2012; Tay et al., 2014; Gola et al., 2018), to elicit high numbers of intrahepatic CD8^+^ T_RM_ cells, and protection against hepatotropic pathogens compared to intradermal and intramuscular vaccine delivery routes (Figure 1) (Epstein et al., 2011; Fernandez-Ruiz et al., 2016; Ishizuka et al., 2016; Gola et al., 2018). In the vagina, several studies suggest that intravaginal delivery of vaccine vectors [HPV PsV, and Ad serotypes 26 (Ad26) and 35 (Ad35)] is the most efficient route to express vaccine-encoded antigens in vaginal tissues and elicit cervicovaginal CD8^+^ T_RM_ cells (Figure 1) (Cuburu et al., 2015, 2018; Fernandez-Ruiz et al., 2016). Furthermore, topical application of chemokine ligands (CXCL9 and CXCL10) in the vagina have been reported to “pull” systemically primed effector CD8^+^ T cells into the vagina and allow these cells to differentiate into CD8^+^ T_RM_ cells (Shin and Iwasaki, 2012).

## Can We Exploit DNA Vaccines to Elicit Tissue-Resident Memory T Cells for Protection Against HIV-1 or HCV?

There has been much research and progress made to improve the immunogenicity of DNA vaccines with respect to the choice of adjuvants, route of vaccine delivery, codon optimization of genes, method of delivery (e.g., electroporation and gene gun), etc. These aspects have been reviewed extensively elsewhere (Nagata et al., 1999; Garmory et al., 2003; Jechlinger, 2006; Vanniasinkam et al., 2006; Jorritsma et al., 2016) and the resulting refinements have led to DNA vaccines being more effectively exploited for use in Phase I and II clinical trials especially in the context of cancer (Kim et al., 2014; Trimble et al., 2015). However, vast majority of the studies including those progressing to the clinic have delivered DNA vaccines using intradermal or intramuscular routes. These routes may not be as effective compared to intravenous route to elicit intrahepatic CD8^+^ T_RM_ cells or the intravaginal route to elicit cervicovaginal CD8^+^ T_RM_ cells (Figure 1).

As mentioned above, it is important that a vaccination regimen designed to elicit CD8^+^ T_RM_ cells facilitate local antigen presentation to naïve and antigen experienced precursors of CD8^+^ T_RM_ cells, which is best achieved by the local expression of vaccine-encoded antigens and/or promoting local inflammation (Figure 1). Manual massaging (Liu et al., 2004), hydrodynamic injections (Yu et al., 2014), and liposome complexes (Kawakami et al., 2000) are some commonly used techniques to transfect hepatocytes *in vivo* following intravenous delivery of DNA. The expression of vaccine-encoded antigens in hepatocytes is a common hallmark of studies that have elicited intrahepatic CD8^+^ T_RM_ cells (Fernandez-Ruiz et al., 2016; Ishizuka et al., 2016), but none of these delivery strategies have led to a licensed vaccine for use in humans. Furthermore, it not known whether any of these strategies can elicit intrahepatic CD8^+^ T_RM_ cells in humans mainly owing to the difficulties of isolating liver biopsies in healthy patients although fine needle aspirates may be used to less invasively sample liver-resident T cells (Gill et al., 2018a,b) and the lack of biomarkers (i.e., in the blood) that can accurately predict the formation of CD8^+^ T_RM_ cells in the liver and other tissues/organs. In the vagina, a proof of concept study has shown that DNA can be expressed following submucosal intravaginal delivery of DNA in mice (Sun et al., 2015). However, the same study reported poorly immunogenic responses in mice and noted that electroporation was required to improve the immunogenicity of intravaginally delivered DNA which could be difficult to exploit in humans.

DNA can be used as a vector to prime high numbers of circulating antigen-specific T cells (Gummow et al., 2015; Wijesundara et al., 2018) which can then be recruited to the liver using vectors that efficiently enter cells in the hepatic tissues or the vagina using chemokine ligands or vectors that transduce vaginal epithelial cells (Figure 1). Furthermore, given the poor transfection efficiency and immunogenicity of DNA when delivered into the vagina or the liver, it is more feasible to exploit DNA as an immune priming agent in a vaccination regimen to elicit HIV-1- or HCV-specific CD8^+^ T_RM_ cells in the vagina or the liver, respectively. Furthermore, analogous strategies using protein-based T cell priming agents in prime/pull (Shin and Iwasaki, 2012), prime/trap (Fernandez-Ruiz et al., 2016), or prime/target (Gola et al., 2018) regimens have been used to elicit protective cervicovaginal or intrahepatic CD8^+^ T_RM_ cells. A caveat in this case is to determine whether the primed T cells express adequate levels of chemokine receptors (e.g., CXCR3 and CXCR6) necessary to home to the liver (Sato et al., 2005; Tse et al., 2014; Gola et al., 2018; Olsen et al., 2018) or the vagina (Shin and Iwasaki, 2012) following DNA immunization. Even if not obligatory, the expression of the relevant homing receptors could be required to ensure that high densities of primed CD8^+^ T cells are recruited to the cervicovaginal mucosa or the liver following introduction of a vaccine vector or an agent (Figure 1) to facilitate the formation of CD8^+^ T_RM_ cells. Several studies have shown that the number of CD8^+^ T_RM_ cells is a crucial parameter that dictates the collective ability of these cells to confer protection against pathogens exposed in the skin, liver, or the vagina with greater numbers favoring protective outcomes (Cuburu et al., 2012; Shin and Iwasaki, 2012; Fernandez-Ruiz et al., 2016; Park et al., 2018).

## Concluding Remarks

DNA has recently re-emerged as an effective vaccination platform in humans, but its use in developing a T cell-based vaccine will likely rely on its ability to be exploited in a regimen that can elicit robust immunity in the vagina and the gut in the context of HIV-1, or the liver in the context of HCV. In this regard, we have highlighted the importance of eliciting cervicovaginal or intrahepatic CD8^+^ T_RM_ cells against these viruses and also reviewed strategies as well as caveats associated with using DNA to elicit localized CD8^+^ T_RM_ cells as a frontline defense against HIV-1 and HCV.

## Author Contributions

DW and ZM conceived the initial drafts of the manuscript. BG-B, MM, AS, CR, RB, AL, and EG revised many parts of the manuscript and contributed to finalize the manuscript.

### Conflict of Interest Statement

The authors declare that the research was conducted in the absence of any commercial or financial relationships that could be construed as a potential conflict of interest.
